# Prenatal Tympanic Ring Anomaly Without Microtia: A Subtle Clue Toward Severe Early‐Onset Monogenic Disorders

**DOI:** 10.1002/pd.70131

**Published:** 2026-03-25

**Authors:** Yung Hang Lam, Mengmeng Shi, Zirui Dong, Kwong Wai Choy, Tak Yeung Leung, Ye Cao

**Affiliations:** ^1^ Private Clinic Central Hong Kong SAR China; ^2^ Department of Obstetrics and Gynaecology The Chinese University of Hong Kong Hong Kong SAR China; ^3^ CUHK‐BCM Joint Centre for Medical Genetics The Chinese University of Hong Kong Hong Kong SAR China

**Keywords:** *ANKRD11*, genome sequencing, hearing loss, microtia, *NIPBL*, prenatal diagnosis, *SOS2*, *TW1ST1*, tympanic ring

## Abstract

**Objective:**

To investigate the genetic etiologies and clinical significance of fetal tympanic ring abnormalities detected during second‐trimester ultrasound in the absence of microtia.

**Method:**

Between November 2019 and June 2024, we examined the fetal tympanic rings of 10,277 unselected pregnant women during the 20–22 weeks of morphology ultrasound. Fetal cases with abnormal tympanic rings, without microtia, underwent amniocentesis for genetic investigation, including chromosome microarray analysis and trio genome sequencing (30×).

**Results:**

Five fetuses (0.05%, 5/10,277) with abnormal tympanic rings in the absence of microtia were identified in this cohort, including three unilaterally (2 right, 1 left) and two bilaterally affected cases. Four fetuses had no other obvious structural abnormalities. One had an isolated unilateral hypoplastic tympanic ring with negative genetic findings and was postnatally found to have conductive hearing loss. The remaining four fetuses were diagnosed with severe early‐onset genetic disorders (80%), including KBG syndrome, Noonan syndrome, Saethre‐Chotzen syndrome, and Cornelia de Lange syndrome. All these four pregnancies were terminated.

**Conclusion:**

Ultrasound evaluation of fetal tympanic ring abnormalities is feasible during the second trimester. Fetal tympanic ring anomalies even occurring with minor ultrasound markers may represent a significant indication for a comprehensive genetic assessment. More studies are needed to establish how tympanic ring anomalies should be integrated into routine clinical practice.

## Key Summary

- What is already known about this topic?
  ◦ The current ISUOG guidelines for the second‐trimester anomaly scan do not include routine examination of the fetal tympanic ring. While studies showed that its evaluation is feasible, abnormality of the tympanic ring is not typically assessed by prenatal imaging. The etiology and outcomes of abnormal fetal tympanic ring remain unclear, particularly in the absence of external ear abnormalities.
- What does this study add?
  ◦ In this study, 80% of fetuses with either hypoplastic or absent tympanic rings in the presence of normal external ears were found to be affected by severe and early‐onset monogenic disorders. Some of these cases presented with only mild concurrent ultrasound abnormalities, including single umbilical artery, accessory auricles, and macrocephaly. These observations highlight that fetal tympanic ring abnormalities, even those occurring with minor ultrasound markers, may represent significant indications for comprehensive genetic assessment. Examination of the external ears alone without the tympanic rings will miss an important clue to the prenatal diagnosis of monogenic Disorders.

## 1 Introduction

The tympanic ring (TR) is a C‐shaped bone structure and provides the osseous framework surrounding the developing tympanic membrane [1]. Over time, this structure progressively integrates into the temporal bone, ultimately becoming the tympanic bone. Embryologically, the tympanic ring plays a crucial role in the induction process of the external auditory meatus formation and mediates the normal development of middle ear structures. There is a strong association between aplasia or hypoplasia of the tympanic ring and conditions such as microtia, congenital aural atresia, and middle ear abnormalities, making the tympanic ring a sensitive marker for congenital hearing loss. Notably, 94% of patients with congenital aural atresia also present with concomitant microtia, though it is rarely associated with inner ear malformations.

The tympanic ring plays a pivotal role during the early development of the external and middle ear, interacting with the ectoderm of the first pharyngeal cleft to initiate a cascade of epithelial–mesenchymal interactions that govern the development of the external auditory canal, tympanic membrane, and malleus handle. Fetal tympanic rings could be firstly seen as distinct rounded regions of mesenchymal condensation in the 8th week of gestation. They begin ossifying over the next 2–3 weeks, and by 19 weeks, the ossification is complete, with the tympanic rings reaching an internal diameter of approximately 7.5 mm. Leibovitz et al. have demonstrated the feasibility of ultrasonographic imaging of fetal tympanic rings during second‐trimester examinations, with a 90% demonstration rate achieved in the 16‐week subgroup [2].

The International Society of Ultrasound in Obstetrics and Gynecology (ISUOG) does not include the fetal ear, including the tympanic rings, as a part of routine assessment in fetal evaluation [3]. Abnormal ear development, whether of the outer or inner structures, is well recognized as being associated with various genetic conditions and etiologies. Ear anomalies, such as microtia, often occur as isolated congenital defects, either unilaterally or bilaterally, but can also present as part of broader genetic syndromes involving craniofacial, renal, or cardiac systems. Some retrospective studies showed that 97% of microtia cases confirmed after birth can be prenatally detected, with 27% complicated with other structural anomalies. Among these, a significant proportion of prenatal microtia cases are associated with chromosomal abnormalities, often in conjunction with other structural anomalies, most commonly cardiac abnormalities [4]. A recent systematic review examining the genetic etiologies of microtia in 1459 patients revealed that in the syndromic microtia group, the most commonly implicated genes were *TCOF1* (43.75%, MIM 606847, Treacher Collins syndrome 1), *SIX2* (4.69%, MIM 604994), and *HSPA9* (4.69%, MIM 604994). In contrast, the non‐syndromic microtia group most frequently identified the *GSC* exon 2 (25%, MIM 138890), followed by *FANCB* (16.67%, MIM 300515) and *HOXA2* (8.33%, MIM 604685) [5]. Thus, the high likelihood of a genetic syndrome underlying fetal ear anomalies highlights the clinical significance of routinely assessing fetal ear development. However, most prenatal and postnatal studies tend to focus primarily on outer ear abnormalities, with a notable lack of research investigating the potential genetic etiologies related to tympanic ring development, an area that remains significantly underexplored.

This study aims to evaluate the significance of prenatal abnormal tympanic rings, either unilateral or bilateral, without microtia. This is the first report evaluating genetic studies of fetuses with abnormal tympanic rings with normal external ears. Our study aims to enhance phenotype‐driven prenatal exome and genome sequencing for precision genetic diagnostics in rare diseases, thereby supporting improved prenatal care.

## 2 Methods

### 2.1 Study Group

This is a retrospective study of all women who attended a private clinic for fetal morphology ultrasound examination at 20–22 weeks of gestational age (GA) between November 2019 and June 2024. In addition to evaluating other fetal structures, bilateral tympanic rings were assessed for all fetuses as part of the ultrasound examination. Hypoplastic tympanic rings were defined when either the transverse diameter or height of the tympanic rings was less than the 1st percentile of the local nomogram and genetic testing including chromosome microarray analysis and genome sequencing (GS) was offered. Fetuses with microtia were excluded from this study. Parental peripheral blood samples were collected to allow trio‐based analyses at the time of prenatal sample retrieval. Written informed patient consents were obtained from all the abnormal cases reported in this study. The genetic study of the amniotic fluid samples was approved by the Joint Chinese University of Hong Kong‐New Territories East Cluster Clinical Research Ethics Committee (CREC no.2016.713).

### 2.2 Fetal Tympanic Ring Sonographic Evaluation

**FIGURE 1:**
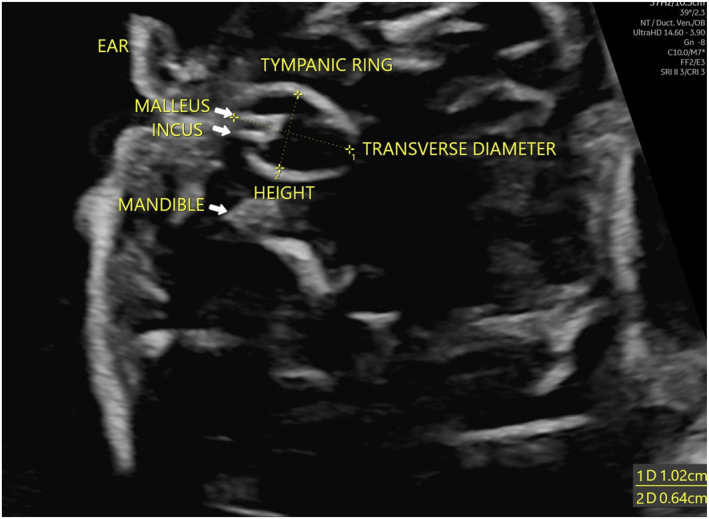
Normal tympanic ring—abdominal 2D ultrasound image of the axial section of the base of the brain to show the tympanic ring, the malleus and incus were in the lateral half of the ring, and the cross‐section of the mandible ramus beneath the ring. The transverse diameter and height were measured by placing the pointers at the inside border of the ring.

The abdominal 2‐D ultrasound examination was performed using a GE Voluson E10 or Expert 22 machine with a C‐2‐9‐D convex probe according to that described by Leibovitz et al. [2]. The preferred position to obtain the best view was when the fetal head was prone, neck flexed and slightly rotated to the side being examined to obtain each side of the tympanic ring one by one rather than obtaining both tympanic rings at the same image. Because of the inclination of the ring, visualization of both tympanic rings in the same plane will not include the entire contour of both rings, particularly the lateral borders. An axial section of the base of the brain at the level around the ear tragus was obtained to show the complete contour of the tympanic ring. On this plane, the longer malleus manubrium can be seen just anterior to the incus at the lateral half of the ring, with the cross‐section of the mandible ramus just beneath the ring (Figure 1). The transverse diameter and height of the tympanic ring were measured by placing the calipers at the inner border of the ring with the radiant mode turned off when employing the Expert 22 machine.

### 2.3 Prenatal Genetic Testing and Data Interpretation

Genomic DNA was extracted directly from uncultured amniotic fluid (AF) samples and parental peripheral blood samples using the DNeasy Blood & Tissue Kit (Cat No./ID: 69,506, Qiagen, Hilden, Germany) for genetic testing. Standard genetic investigations quantitative fluorescent polymerase chain reaction (QF‐PCR) of short tandem repeat markers excluded maternal cell contamination. Chromosomal microarray analysis and genome sequencing were performed. Chromosomal microarray analysis (CMA) was performed using FetalDNA Chip v2.0 (8 × 60 K, in‐house designed aCGH + SNP array, Agilent Technologies, https://www.obg.cuhk.edu.hk/services/laboratoryservices/chromosomal‐microarray‐analysis/), and data analysis was performed with CytoGenomics software according to the manufacturer's protocol [6].

Genome sequencing (GS, 30×) was performed after CMA results were available, using the PCR‐free library preparation kit (MGI Tech Co. Ltd.). The libraries were sequenced on an MGISEQ‐2000 platform for over 400 million read‐pairs with 150‐bp in length per case, equivalent to sequencing read‐depths of > 30× per case. Genomic variants were detected using both well‐established publicly available pipelines and our in‐house bioinformatics analysis pipelines for SNVs, InDels, CNVs (read‐depth‐based detection and chimeric‐read‐based detection), structural rearrangements (including translocations, inversions and insertions by chimeric‐read‐based detection), and absence of heterozygosity by detection of variant allelic fractions. Both the CNV detection and structural rearrangement detection algorithms used in this analysis were sensitive to genetic aberrations that were beyond the resolution limit of CMA and karyotyping, respectively. The sequencing approach and data analysis were according to the previously published studies, following the published protocols [7, 8, 9, 10, 11, 12, 13]. The variants were integrated for interpretation in accordance with the guidelines of the American College of Medical Genetics and Genomics (ACMG) and the Association for Molecular Pathology (AMP) [14, 15]. Pathogenic or likely pathogenic (P/LP) variants which explained or partially explained the presenting fetal phenotype were considered diagnostic variants. Incidental findings were reported according to the ACMG recommendations in reporting secondary findings in CMA and genome sequencing. Genetic variants in the gene list of ACMG SF (v3.0 before 2024, or v3.2 after 2024) that do not explain the phenotype of the fetus, albeit potentially pathogenic, were reported as medically actionable variants [16, 17]. If the parents had opted in to receive this information in pretesting consents, the P/LP variants of these recommended conditions were reported.

### 2.4 Statistical Analysis

In the present study, measurements of the transverse diameter and height were obtained from 200 tympanic rings derived from 100 fetuses at GA of 20–20 weeks and 6 days. The normality of the distribution for both parameters was assessed using the Jarque‐Bera test. *p*‐value ≥ 0.05 will be accepted as a normal distribution.

## 3 Results

### 3.1 Nomogram of Fetal Tympanic Ring

**FIGURE 2:**
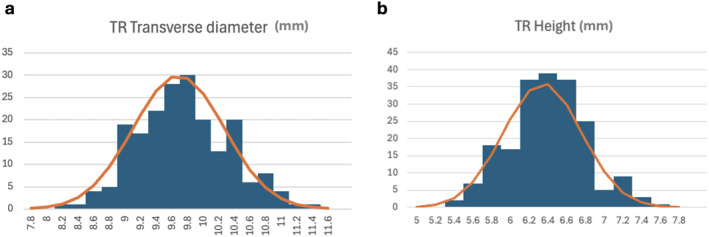
Distribution of measurements for the tympanic ring transverse diameter (a) and height (b). The *Y* axis represents the counting of tympanic rings. The *X* axis represents the measurements (mm) for the transverse diameter and height of tympanic rings.

A local nomogram was established from 200 tympanic rings (100 left and 100 right) in one hundred fetuses at the gestational age of 20–20 weeks and 6 days (Table S1). The transverse diameters of the tympanic ring ranged from 8.2 to 11.4 mm, and the data exhibited a normal distribution, validated by the Jarque‐Bera test (*p* = 0.448) (Figure 2a). The height of the tympanic ring ranged from 5.4 to 7.5 mm, and it also showed a normal distribution, as confirmed by the Jarque‐Bera test (*p* = 0.064) (Figure 2b). The mean, 5th percentile, and 1^st^ percentile of transverse diameter and height were 9.7 × 6.3, 8.8 × 5.7, 8.3 × 5.4 mm. The hypoplastic tympanic ring was defined when either of these measurements was less than the 1st percentile.

### 3.2 Prevalence

During the study period, a total of 10,277 patients and 20,554 tympanic rings were examined; the majority came for routine fetal morphology screenings, while a minority were referred due to suspected fetal anomalies. In some cases, reassessment was successfully performed a few days later due to unfavorable fetal positioning.

Overall, twelve cases (0.12%, 12/10,277) of 17 hypoplastic or absent tympanic rings were identified in this cohort, which estimated the overall frequency of abnormal tympanic ring be approximately 0.08% (17/20,544) in the prenatal general population. Seven cases with concurrent grade 1–3 microtia were excluded, leaving five fetuses with seven abnormal tympanic rings (0.05%, 5/10,277; Table 1) in the absence of microtia for inclusion in this study. Consequently, the incidence of tympanic ring anomalies in the absence of microtia in this cohort was 0.03% (7/20,554) with no lateral preference (3 right‐sided and 4 left‐sided).

**TABLE 1:** The clinical and genetic data of the five fetuses with abnormal tympanic rings without microtia.

Case	Maternal age (year)	GA (week + day)	Tympanic ring Abnormality (dimensions)	Other ultrasound findings	CMA results	Genome Sequencing Results	Pathogenicity & inheritance	Reported (PMID);/novel ACMG classification	Related disorder, OMIM	Outcome
1	30	21 + 1	Left hypoplastic (6.9 × 3.9 mm)	Not detected	Negative	Negative	Not applicable	N.A.	Not applicable	Liveborn with left sided conductive deafness
2	32	20 + 4	Right hypoplastic (7.6 × 3.7 mm)	Single umbilical Artery	Negative	Heterozygous *ANKRD11* (NM_013275.6):c.2177_2178del p.(Lys726ArgfsTer15)	Pathogenic, de n*ovo*, Autosomal dominant	Reported (PMID; 35970914) Pathogenic, PVS1+PS2+PM2	KBG syndrome, # 148050,	Terminated
3	32	20 + 5	Right absent	Accessory auricle	Negative	Heterozygous *SOS2* (NM_006939.4):c.1385T > C p.(Phe462Ser)	Likely pathogenic, *de novo*, Autosomal dominant	Novel Likely pathogenic, PS2+PM2+PP3	Noonan syndrome 9, # 616559	Terminated
4	32	22 + 2	Bilateral hypoplastic (left 7.9 × 4.8 mm) (Right 7.6 × 4.0 mm)	Macrocephaly	Negative	Heterozygous *TWIST1* (NM_000474.4):c.475_477dup p.(Leu159dup)	Likely pathogenic, de n*ovo*, Autosomal dominant	Novel Likely pathogenic PS2+PM2	Saethre‐chotzen syndrome with or without eyelid anomalies,# 101400	Terminated
5	35	21 + 2	Bilateral absent	Tetralogy of fallot, Retro‐esophageal brachiocephalic vein, Cleft palate, Short limbs	Negative	Heterozygous *NIPBL*(NM_133433.4):c.5167C > T p.(Arg1723Ter)	Pathogenic, *de novo*, Autosomal dominant	Reported (PMID: 15318302) Pathogenic, PVS1+PS2+PM2	Cornelia de Lange syndrome 1, # 122470	Terminated

Abbreviations: CMA, chromosomal microarray analysis; GA, gestational age; N.A., not available.

These five cases were detected at a mean gestational age of 21 + 1 week (from 20 + 4 to 22 + 2 weeks). Among them, three cases were unilaterally affected, two cases were bilaterally affected, and Case 5 exhibited bilateral, completely absent tympanic rings. Case 1 only had an isolated unilateral left hypoplastic tympanic ring. Case 2–5 presented with additional minor or severe abnormalities, including the single umbilical artery, accessory auricle, macrocephaly, tetralogy of Fallot, retroesophageal brachiocephalic vein, cleft palate, and short limbs.

### 3.3 Genetic Investigations

**FIGURE 3:**
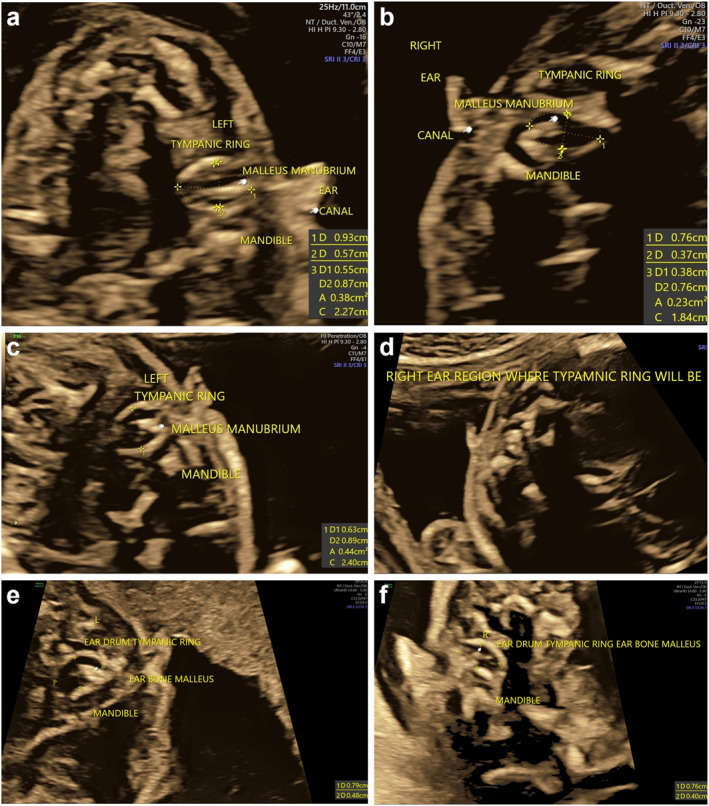
Fetal tympanic ring anomalies in cases 2–4. (a,b) Case 2, unilateral right hypoplastic tympanic with KBG syndrome. (c,d) Case 3, unilateral absent right tympanic ring with noonan syndrome. (e,f) Case 4, bilateral hypoplastic tympanic rings with saethre‐chotzen syndrome; L, Left; R, Right.

All these five cases were submitted for standard genetic investigations. Chromosome microarray analysis (CMA) results were negative in all cases, whereas genome sequencing identified pathogenic or likely pathogenic variants in four fetuses (Cases 2–5). All these diagnostic findings occurred *de novo* and were associated with severe autosomal dominant disorders, including KBG syndrome, Noonan syndrome, Saethre‐Chotzen syndrome, and Cornelia de Lange syndrome. Figure 3 shows the abnormal tympanic rings in Cases 2 (Figure 3a,b), 3 (Figure 3c,d) and 4 (Figure 3e,f). For the four genetically diagnosed cases, pregnancies were terminated, with an expected low recurrence risk unless germline mosaicism is present. Case 1 was carried to term. Postnatal follow‐up revealed conductive hearing loss but otherwise normal external ear and external auditory meatus and normal development.

## 4 Discussion

We demonstrated that the tympanic ring can be consistently visualized and examined during the 20‐week fetal morphology ultrasound scan. Previous observations showed a strong association between microtia and tympanic ring anomalies [2]. In another study from China of 18 fetuses (19 ears) with microtia, dysplastic or hypoplastic tympanic rings were associated with aural atresia [18]. This study highlights that tympanic ring anomalies may occur independently of microtia. Isolated tympanic ring abnormalities, as observed in Case 1, may serve as early predictors of aural atresia and conductive hearing loss postnatally. When combined with other ultrasound abnormalities, they may elevate the chance of genetic defects. This is understandable because 30% of genetic cases of sensorineural hearing loss are syndromic [19]. Notably, in our series, Case 2–4 only exhibited mild additional features, which might otherwise be dismissed as having low genetic significance. For instance, the single umbilical artery in Case 2 would be frequently considered a soft marker [20], and the isolated accessory auricles in Case 3 are often considered to have only cosmetic implications. Without concurrent tympanic ring assessment, these cases could evade genetic investigation, delaying critical diagnoses. Detection of a unilateral abnormal tympanic ring is easier because of the presence of the normal side for comparison, but Case 4 demonstrated that bilateral hypoplastic tympanic rings can also be detected with systematic imaging (Figure 3e,f). In summary, ultrasound examination of the fetal tympanic ring at the 20‐week morphology scan is feasible, and this provides valuable information as an isolated abnormality and in cases with other abnormal ultrasound features, including mild variants.

In this study, we arbitrarily used the 1st percentile as the cutoff because at the start we do not know the clinical implications of a borderlinely small tympanic ring. To avoid creating unnecessary anxiety, we only inform the patients of the extreme cases. With more experience, we think that a practical, easy lower cutoff of tympanic transverse diameter and height at 20 weeks will be 8 mm, and 5 mm. In the report by Wang et al. [20], they also found that tympanic ring dimension less than the 1^st^ percentile was associated with external auditory meatus stenosis.

The most common syndromes associated with microtia are reported as oculoauriculovertebral spectrum (OAVS), Goldenhar syndrome (GS)/hemifacial microsomia/craniofacial microsomia (CFM), Treacher Collins, Nager, DiGeorge, and CHARGE syndrome [21]. More than 20 genes have been identified in human Mendelian syndromes with microtia as a major feature [22]. Interestingly, this cohort with hypoplastic tympanic rings without microtia on prenatal ultrasound implicated a distinct group of genetic syndromes, including KBG syndrome, Noonan syndrome, Saethre‐Chotzen syndrome, and Cornelia de Lange syndrome. This divergence suggests that different developmental mechanisms and regulatory pathways may be involved. Classic presentations of these syndromes showed very distinct dysmorphic features, while interestingly, these syndromes are commonly reported with conductive hearing loss [23, 24, 25, 26].

KBG syndrome [MIM:148050] is characterized by macrodontia, characteristic facial features, short to normal stature, developmental delay, or intellectual disability. It also presents with a characteristic audiological profile, including conductive hearing loss (71%), bilateral ear involvement (81%), mild to moderate severity (84%), and stable progression (69%), although some audiological heterogeneity is noted [27]. Among patients with abnormalities on CT imaging (55%), the most common findings were ossicular chain impairment (67%), fixation of the stapes footplate (33%), and inner ear malformations (33%). According to Bianchi's review, hearing loss is typically conductive and may be the first sign of KBG syndrome, sometimes identified through speech delay or after a normal neonatal hearing screening [24]. Published cases of SOS2‐related Noonan syndrome 9 [MIM:616559] are limited. However, dysmorphic external ear anomalies, along with both sensorineural and conductive hearing impairment, are commonly reported in a cohort of 97 clinically diagnosed Noonan syndrome patients, 79% of whom had a genetic diagnosis. External ear anomalies were found in 77% of Noonan syndrome patients, including low‐set or posteriorly rotated ears. Interestingly, 9% of this cohort showed variable sensorineural hearing impairment, ranging from mild to profound, 2% experienced permanent conductive hearing loss, and 2% had mixed hearing loss. Notably, 20% of these patients exhibited temporary conductive hearing impairment due to otitis media with effusion, which resolved between the ages of 2 and 18 years [23] Saethre‐Chotzen Syndrome [MIM:101400] is characterized by coronal synostosis, facial asymmetry, strabismus, ptosis, and characteristic ear features such as a small pinna with a prominent superior and/or inferior crus. For children with *TWIST1*‐associated Saethre‐Chotzen Syndrome, hearing loss is common (80%), with at least 50% of patients having confirmed conductive hearing loss associated with middle ear effusion [26]. Cornelia de Lange syndrome [CdLS, MIM: 122470] is characterized by distinctive facial features, prenatal growth restriction, short stature and < 5th centile throughout life, hypertrichosis, and upper‐limb reduction defects that range from subtle phalangeal abnormalities to oligodactyly. Hearing loss is very common (85%–90%) in individuals with CdLS. It is typically bilateral, present in infancy, and ranges from mild to severe (40%–50%) and is sensorineural in ∼25% and conductive in 60%–75% [25, 28]. More than 50% of the patients seen in an adult CdLS clinic reported improvement in hearing loss over time [29].

More importantly, while hearing loss is not the most critical symptom in these syndromes, developmental delay and intellectual disability often are the key concerns of the parents. These symptoms are usually not detectable or measurable through prenatal ultrasound. However, the presence of abnormal tympanic rings could serve as a crucial indicator of the underlying syndromes, prompting comprehensive genetic evaluations. Negative results for common genetic disorders in such cases may indicate a favorable outcome, highlighting the prognostic significance of ultrasound assessment of the tympanic ring.

This is a single‐center study conducted primarily in a mainly Chinese population, which may limit generalizability to other ethnic groups. Additionally, the sample size was small, and further multicenter validation is needed to confirm our findings. We also anticipate that other monogenic disorders may be present in cases without abnormal tympanic rings, considering the prevalence of such rare genetic disorders in the population. However, to our knowledge, this is the first study to examine the prenatal genetic correlation between tympanic ring anomalies in the absence of microtia and syndromic conditions through genome sequencing. Our findings highlight that tympanic ring abnormalities may serve as subclinical indicators for syndromes characterized by intellectual disability or developmental delay that typically manifest postnatally, thereby offering a potential early prenatal marker for targeted genetic counseling and management.

## 5 Conclusion

In conclusion, we demonstrate that systematic evaluation of the fetal tympanic ring during routine mid‐trimester ultrasound is feasible and clinically significant. Even in the absence of microtia, tympanic ring anomalies may predict conductive hearing loss. When observed together in the presence of other ultrasound markers, it may indicate a serious monogenic disorder. Further studies are needed for a more comprehensive understanding of the use of routine ultrasound examination of fetal tympanic rings in the prediction of aural atresia and its association with genetic disorders.

## Funding

National Key Research and Development Program of China (Project 2019YFE0198300) to Kwong Wai Choy and Ye Cao; The Chinese University of Hong Kong, Direct Grant for Research (Reference No.: 2025.164) to Ye Cao; The Chinese University of Hong Kong, Direct Grant for Research (Reference No.: 2024.157) to Kwong Wai Choy and Mengmeng Shi.

## Ethics Statement

The genetic study of the amniotic fluid samples was approved by the Joint Chinese University of Hong Kong‐New Territories East Cluster Clinical Research Ethics Committee (CREC no.2016.713).

## Consent

Written informed patient consent was obtained from all the abnormal cases reported in this study.

## Conflicts of Interest

Dr. Yung Hang Lam is the owner of a private clinic in Central.

## Supporting information


**Table S1:** Measurement details of 200 tympanic rings (100 left and 100 right) from 100 fetuses.

## Data Availability

The data are not publicly available because public access was not provided by the subjects in the study, but could be available upon reasonable request from the corresponding author.
